# Perforin and IL-2 Upregulation Define Qualitative Differences among Highly Functional Virus-Specific Human CD8^+^ T Cells

**DOI:** 10.1371/journal.ppat.1000798

**Published:** 2010-03-05

**Authors:** George Makedonas, Natalie Hutnick, Danielle Haney, Alexandra C. Amick, Jay Gardner, Gabriela Cosma, Adam R. Hersperger, Douglas Dolfi, E. John Wherry, Guido Ferrari, Michael R. Betts

**Affiliations:** 1 Department of Microbiology, University of Pennsylvania, Philadelphia, Pennsylvania, United States of America; 2 The Wistar Institute, Philadelphia, Pennsylvania, United States of America; 3 Department of Surgical Sciences, Duke University Medical Center, Durham, North Carolina, United States of America; NIH/NIAID, United States of America

## Abstract

The prevailing paradigm of T lymphocyte control of viral replication is that the protective capacity of virus-specific CD8^+^ T cells is directly proportional to the number of functions they can perform, with IL-2 production capacity considered critical. Having recently defined rapid perforin upregulation as a novel effector function of antigen-specific CD8^+^ T cells, here we sought to determine whether new perforin production is a component of polyfunctional CD8^+^ T cell responses that contributes to the control of several human viral infections: cytomegalovirus (CMV), Epstein-Barr virus (EBV), influenza (flu), and adenovirus (Ad). We stimulated normal human donor PBMC with synthetic peptides whose amino acid sequences correspond to defined CTL epitopes in the aforementioned viruses, and then used polychromatic flow cytometry to measure the functional capacity and the phenotype of the responding CD8^+^ T cells. While EBV and flu-specific CD8^+^ T cells rarely upregulate perforin, CMV-specific cells often do and Ad stimulates an exceptionally strong perforin response. The differential propensity of CD8^+^ T cells to produce either IL-2 or perforin is in part related to levels of CD28 and the transcription factor T-bet, as CD8^+^ T cells that rapidly upregulate perforin harbor high levels of T-bet and those producing IL-2 express high amounts of CD28. Thus, “polyfunctional” profiling of antigen-specific CD8^+^ T cells must not be limited to simply the number of functions the cell can perform, or one particular memory phenotype, but should actually define which combinations of memory markers and functions are relevant in each pathogenic context.

## Author Summary

Although CD8^+^ T cells are thought to be largely responsible for the control of viral infections, exactly how they mediate protection is uncertain. One approach to assessing their protective capacity is to measure several of their functions simultaneously. Generally, it is believed the more functions a cell can perform, the better its potential to control viral replication. A multi-functional response including interleukin-2 (IL-2) production is currently valued as the key correlate of protection. We recently characterized a novel CD8^+^ T cell function: rapid perforin upregulation, which serves to contribute to and sustain the killing of virally infected host cells. In this study, we show that new perforin is abundant during adenovirus and cytomegalovirus infections, but scarcely detected in the context of influenza and Epstein-Barr virus. Importantly, perforin and IL-2 are rarely co-expressed. The significance of this relationship is that we can no longer assume the more functions a CD8^+^ T cell performs in response to a virus the better. Thus, when considering vaccine design, no single functional profile will likely be protective across all pathogens. Rather, vaccine-induced T cell responses may need to be “pathogen-specific”, as different T cell functional responses will be important for controlling different viral infections.

## Introduction

Understanding the mechanisms by which human T cells provide effective control of pathogens is important for designing interventions against those that persist to cause severe morbidity and/or mortality. T cells generally limit the replication of Epstein Barr virus (EBV)[1],[2], Cytomegalovirus (CMV)[3],[4],[5], and Hepatitis viruses B[6],[7],[8] and C[9],[10], but only rarely of the Human Immunodeficiency Virus (HIV), as the majority of HIV infections inevitably result in progressive disease. Cytotoxic T lymphocytes (CTL) are thought to be a primary mediator of viral control, due in large part to their ability to recognize and eliminate virally infected autologous cells. Although CD8^+^ T cells respond to viral infection with a plethora of effector functions, the identification of a definite immune correlate of protection has not been forthcoming for any human pathogen.

Recent strategies of assessing human antiviral T cell responses focus on the quality of the T cell response, defined by its polyfunctional nature. Briefly, the more effector functions that constitute the overall response, the more protective the response is considered[11],[12]. Typically, the functions quantified simultaneously include upregulation of interferon gamma (IFN-γ) and interleukin-2 (IL-2)[13],[14],[15]. A more elaborate assessment of the T cell response may include a measurement of tumour necrosis factor alpha (TNF-α), a chemokine such as MIP-1β, and degranulation measured by CD107a exposure. A high frequency, multi-functional CD4^+^ T cell response composed of IFN-γ, IL-2, and TNF-α provides protection against Leishmania major infection in mice[16], however a similar correlation in humans for antiviral CD8^+^ T cells has not been formally proven. This is likely because none, or any combination, of these functions may directly inhibit pathogen replication.

CTL clear virally infected target cells primarily via the exocytosis of cytotoxic granules containing granzymes and perforin[17],[18],[19],[20]. The manifestations of genetic mutation or deletion of perforin are impaired cellular cytotoxicity and profound immunodeficiency[21],[22]. We have recently shown that human CD8^+^ T cells can rapidly upregulate perforin *de novo* after antigen-specific stimulation[23], which is immediately transported to the immunological synapse where it likely potentiates cytotoxicity[24]. The measurement of new perforin is different from that of pre-formed perforin stored in cytotoxic granules, in that it indicates the potential of the cell to rapidly reconstitute its cytotoxic nature. In contrast, the assessment of pre-formed perforin in granules indicates immediate killing potential, but likely does not predict the sustainability of the cytotoxic response. Thus, analyzing this novel aspect of T cell functionality could provide new insight into how CD8^+^ T cells mediate pathogenic control.

Here we examine perforin upregulation ability in the context of polyfunctional CD8^+^ T cell responses to several common human viral pathogens: Cytomegalovirus (CMV), Epstein-Barr virus (EBV), Adenovirus (Ad), or Influenza (flu). Infection by EBV, CMV, flu, or Ad stimulates robust memory T cell responses that are associated with protection from viral pathogenesis. However, each course of infection is different: CMV establishes latency but remains lytically active, thereby creating a constant supply of antigen to the immune system, whereas EBV enters the lytic phase infrequently after establishing latency. Thus, EBV-specific CD8^+^ T cells likely only receive periodic restimulation. Primary Ad and flu infections are quickly resolved by the host immune response, but since Ad may become persistent, and there are many Ad serotypes whose sequences are highly conserved[25],[26], Ad-specific CD8^+^ T cells are likely repeatedly stimulated. In contrast, flu infections are seasonal and readily cleared, thus flu-specific CD8^+^ T cell restimulation is likely more intermittent than that for other viruses. We show that the measurement of perforin upregulation redefines our interpretation of polyfunctional CD8^+^ T cell responses and memory phenotypes that we associate with control of these pathogens, and represents a novel correlate of antiviral immunity that should be considered in assessments of human antiviral CD8^+^ T cell responses.

## Results

**Figure 1:**
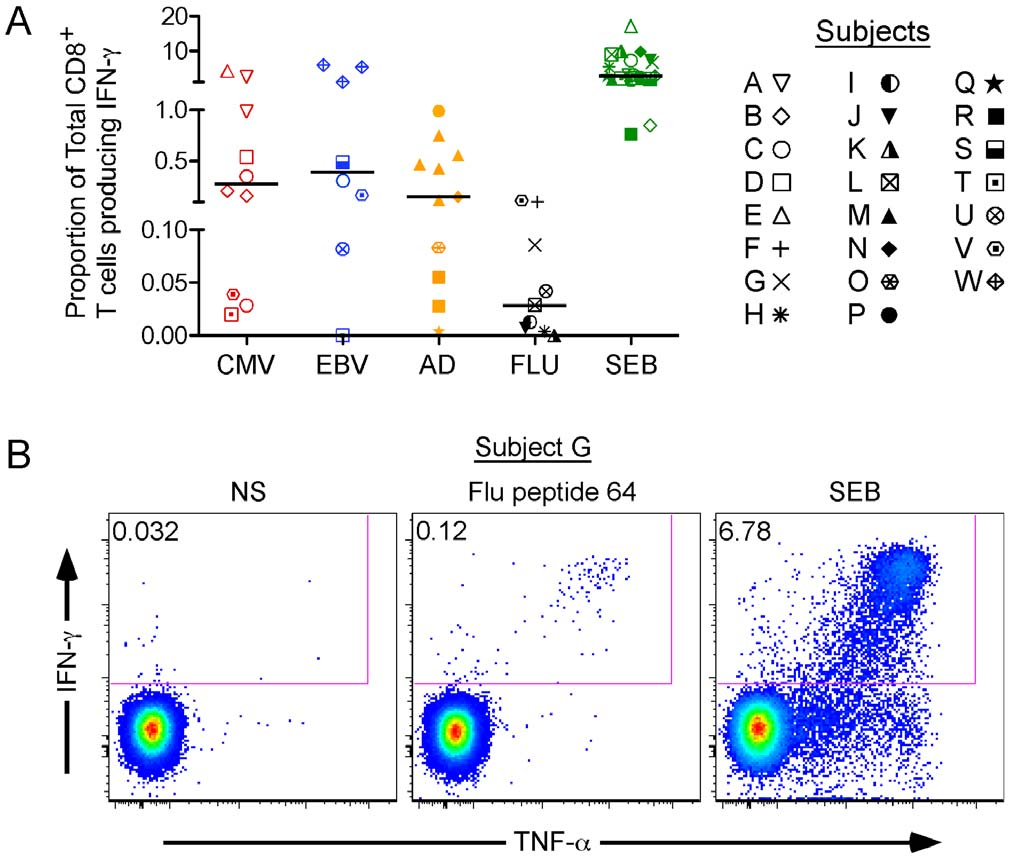
IFN-γ production as a basal marker of activation for antiviral CD8^+^ T cells. (A) IFN-γ production in response to viral peptides. Cells from 23 normal human donors (symbols for each donor are listed in the legend) to produce IFN-γ in response to stimulation with peptide antigens from four different viruses: cytomegalovirus (CMV), Epstein-Barr virus (EBV), Adenovirus (Ad), and influenza (Flu). The responses are grouped by viral specificity: CMV in red, EBV in blue, Ad in orange, and flu in black. The superantigen Staphylococcus Enterotoxin B (SEB) was used as a positive control (green symbols). Some subjects were screened with more than one stimulus (see Table 1). For example, subject C was stimulated with 3 peptides, hence 3 open circles on the graph. The black horizontal bars indicate the median response in each group. (B) A representative CD8^+^ T cell IFN-γ response, after stimulation with flu peptide 64 in Donor G. Unstimulated (NS) and SEB-stimulated cells are shown as negative and positive controls, respectively.

We assayed 23 normal donors for memory CD8^+^ T cell responses against CMV, EBV, Ad, or flu, as defined by the ability to upregulate IFN-γ, TNF-α, IL-2, and/or perforin, or to degranulate, in response to stimulation with individual or pools of synthetic peptides that represent defined CTL epitope(s) in the amino acid sequence of the corresponding virus (Table 1). Individual peptide stimuli were determined by prior epitope mapping experiments, whereas pools of peptides were used on subjects for whom specific epitopes were not identified. We used IFN-γ production as a basal readout of activation; as shown in Figure 1A, we were able to detect CD8^+^ T cell responses against at least one viral peptide or peptide pool in each of the 23 donors. Two individuals did not produce IFN-γ in response to peptide stimulation (1 EBV, 1 flu) but instead produced TNF-α and/or IL-2 (data not shown). The largest virus specific responses we noted were CMV- or EBV-specific, with some individuals exhibiting responses up to ∼5% of total CD8^+^ T cells against a single epitope [median (black bar on Figure 1A) CMV = 0.28%, median EBV = 0.40%]. Ad-specific responses were found up to ∼1% of total CD8^+^ T cells against pools of hexon or E1A-derived peptides (median = 0.15%). Flu-specific IFN-γ production tended to be of lower magnitude than that for other viruses (median flu = 0.029%). Despite the low magnitude, flu-specific IFN-γ responses were readily detectable (Figure 1B). All subjects produced strong CD8^+^ T cell IFN-γ responses after SEB stimulation (Figure 1A, median = 2.76%).

**Table 1:** Peptides used as stimuli for each subject.

Subject	Stimulus	Amino Acid Sequence
A	CMV (pp65)	SDEEEAIVAYTL
	CMV (pp65)	TPRVTGGGAM
B	CMV (pp65)	NLVPMVATV
	CMV (pp65)	IPSINVHHY
C	EBV (BMLF1)	GLCTLVAML
	CMV (pp65)	NLVPMVATV
	CMV (pp65)	RKTPRVTGGGAMAGA
D	CMV (pool)	37 peptides
E	CMV (pp65)	RKTPRVTGGGAMAGA
F	Flu (NP)	RIAYERMCNILKGKF
G	Flu (NP)	IRPNENPAHKSQLVM
H	Flu (Matrix 1)	GILGFVFTL
I	Flu (NP)	RASVGKMIGGIGRFY
J	Flu (Matrix 1)	AVKLYRKLKREITFH
K	Flu (NP)	RIAYERMCNILKGKF
L	Flu (Matrix 1)	TKGILGFVFTLTVPS
M	Ad Hu5 Hexon (CR1 pool)	43 peptides
	Ad Hu5 Hexon (CR2 pool)	41 peptides
	Ad Hu5 Hexon (CR3 pool)	49 peptides
N	Ad Hu5 Hexon (CR2 pool)	41 peptides
O	Ad Hu5 Hexon (CR2 pool)	41 peptides
P	Ad Hu5 Hexon (CR1 pool)	43 peptides
	Ad Hu5 Hexon (CR2 pool)	41 peptides
	Ad Hu5 Hexon (CR3 pool)	49 peptides
Q	Ad Hu5 Hexon (CR2 pool)	41 peptides
R	Ad Hu5 E1a (pool)	46 peptides
	Ad Hu5 Hexon (VR pool)	36 peptides
S	EBV (BZLF1)	RAKFKQLL
T	CMV (pp65)	RKTPRVTGGGAMAGA
	EBV (EBNA3A)	RPPIFIRRL
U	Flu (NP)	ELRSRYWAI
	EBV (BZLF1)	RAKFKQLL
V	Flu (Matrix 1)	GILGFVFTL
	EBV (EBNA3A)	YPLHEQHGM
	CMV (pp65)	NLVPMVATV
W	EBV (BZLF1)	RAKFKQLL
	EBV (EBNA3A)	FLRGRAYGL
	EBV (EBNA3A)	QAKWRLQTL

### IFN-γ producing virus-specific CD8^+^ T cells differentially express other functions

While IFN-γ production is commonly measured to identify virus-specific CD8^+^ T cells, it is unclear whether or not it represents a true correlate of immune protection for EBV, CMV, flu, or Ad. We therefore assessed the capacity of IFN-γ producing CD8^+^ T cells to perform other functions which might be associated with viral control, including rapid perforin upregulation, TNF-α, IL-2, and degranulation.

**Figure 2:**
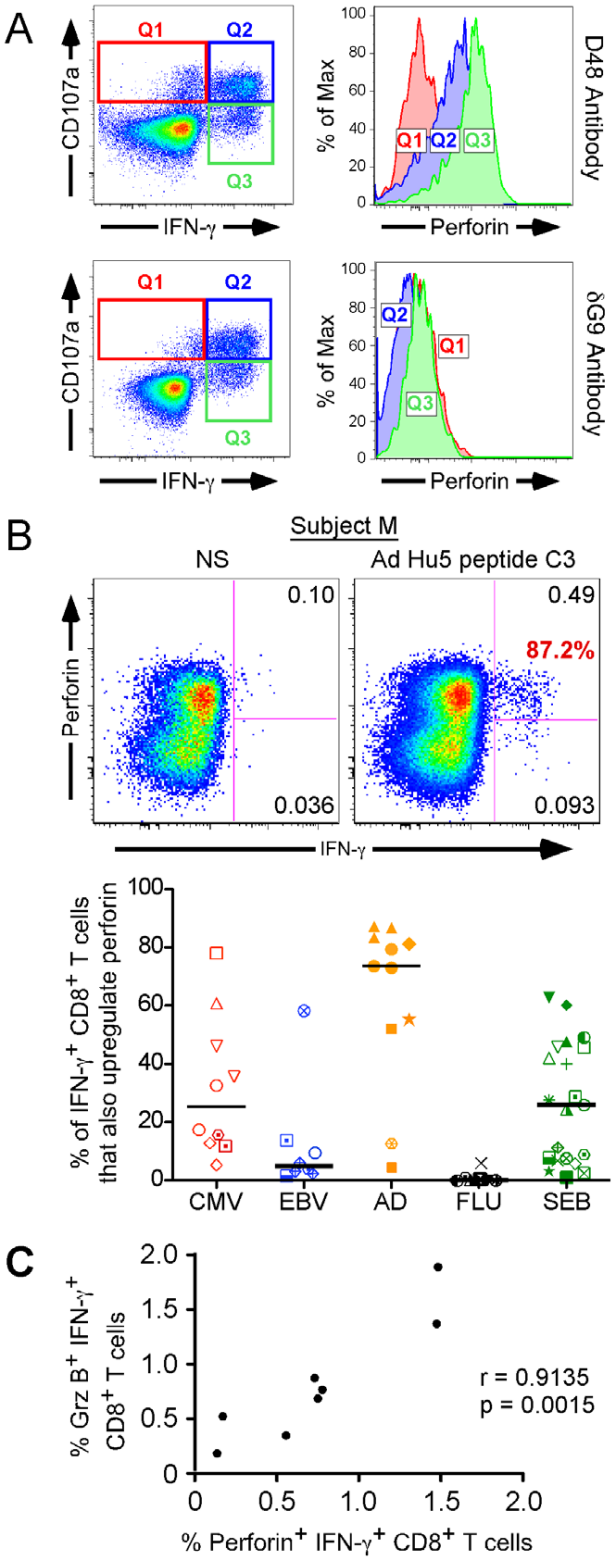
Perforin upregulation by CD8^+^ T cells after peptide-specific activation. (A) Detection of Donor E perforin upregulation in the context of degranulation and IFN-γ production. The dot plots in the left panels define the IFN-γ and CD107a functional subsets (Quadrants 1–3), while the histograms on the right illustrate the perforin content of each functional subset. The cells shown in the top row were stained with the anti-perforin antibody clone D48, whereas those in the bottom row were stained with the δG9 perforin antibody. The histograms depict the perforin content of the corresponding quadrants, as matched by colour. For example, the red histogram represents the perforin content of Quadrant 1 (Q1), defined by the red box on the dot plot. (B) A representative example of perforin upregulation by IFN-γ producing CD8^+^ T cells, in response to Ad Hu5 peptide C3 is shown in the left panel. The value in red represents the proportion of IFN-γ producing CD8^+^ T cells that also produce perforin, whereas the black quadrant numbers reflect the proportion of total CD8^+^ T cells producing the given function. NS =  no stimulation. The right panel illustrates perforin upregulation in IFN-γ^+^ CD8^+^ T cells after activation with peptides from CMV (red), EBV (blue), Ad (orange), and flu (black) by the cohort. Individual symbols represent different subjects (see Figure 1). The black horizontal bars indicate the median response in each group. (C) Perforin and granzyme B are co-expressed in activated CD8^+^ T cells. The proportion of CD8^+^ T cells making new perforin in response to SEB stimulation was plotted against that of CD8^+^ T cells making granzyme B for 8 subjects. The values for both granzyme B and perforin represent cells that were first defined as IFN-γ^+^. The significance of the relationship was determined by calculating the Pearson product-moment correlation coefficient, r.

We recently characterized rapid perforin upregulation as a novel function of antigen-specific CD8^+^ T cells[24], the measurement of which indicates the cells' potential to sustain cytotoxicity. Briefly, newly produced perforin can be detected by a specific anti-perforin antibody (clone D48) which recognizes both pre-formed perforin stored in cytotoxic granules as well as new perforin that has been rapidly produced in response to antigenic stimulation. In contrast, a second perforin antibody (clone δG9) primarily recognizes perforin within cytotoxic granules. By quantifying perforin with the D48 antibody together with another function such as IFN-γ, it is possible to discriminate new perforin from pre-formed granule-associated perforin in activated CD8^+^ T cells. As shown in Figure 2A (top row), the activated cells that produced IFN-γ but failed to degranulate possessed the most perforin (Q3, green). Activated CD8^+^ T cells that both degranulated and upregulated IFN-γ harbored an intermediate amount of perforin (Q2, blue). Perforin was essentially absent in degranulating cells that failed to also produce IFN-γ (Q1, red). The Q1 and Q2 populations were both degranulating to the same degree, yet they represent responding cells that differentially upregulate perforin production. This may simply be an issue of kinetics, in that the cells that only degranulate may not have yet upregulated perforin, or signify a truly separate subpopulation that cannot upregulate perforin. Similarly, the IFN-γ producing responder population (Q2 and Q3) is divided into distinct functional (perforin ± CD107a) subsets. In contrast, the δG9 antibody, specific only for granule-associated perforin, failed to detect perforin in any of the functional subpopulations (Figure 2A, bottom row). Together, these data indicate that antigen-specific human CD8^+^ T cells are capable of upregulating perforin rapidly after stimulation, in the absence of cellular proliferation, and without the addition of exogenous cytokines or other co-factors.

We next examined whether rapid perforin upregulation was characteristic of EBV, CMV, flu, and Ad-specific CD8^+^ T cell responses in our subject cohort. First, we determined what proportion of every antigen-specific IFN-γ response also upregulated perforin in each donor. A representative example is shown in Figure 2B (left panel), where 87.2% of the IFN-γ producing cells concomitantly upregulated perforin. The cohort results are illustrated in the right panel of Figure 2B. Whereas nearly all IFN-γ^+^ Ad-specific CD8^+^ T cells upregulated perforin [orange group, median (black bar) = 73.6%], those responding to EBV and flu displayed limited perforin upregulation [blue group, median = 4.95% and black group, median = 0%, respectively]. Only donor U mounted a substantial EBV-specific perforin response (58.3% of the IFN-γ^+^ CD8^+^ T cells). CMV-specific perforin upregulation was highly variable between subjects [red group, median (black bar) = 25.1%, range: 5.39%–78.0%]; while some donors exhibited strong perforin upregulation (donors D = 78.0% and E = 60.9%), others were more limited [donor B, red open diamond: CMV peptide 20 = 5.39%, CMV peptide 23 = 12.8%]. Polyclonal SEB stimulation also resulted in varying degrees of perforin responsiveness [green group, median = 26.0%, range: 1.02%–62.8%]. Thus, immediate perforin upregulation is not an effector function common to all antigen-specific CD8^+^ T cells; rather it seems to be characteristic of CD8^+^ T cells specific for particular viral infections.

Since perforin is typically expressed with other cytotoxic proteins[19],[27],[28], we assayed for concomitant granzyme B and perforin upregulation in response to SEB stimulation in a small cohort of normal PBMC donors. As depicted in Figure 2C, perforin and granzyme B upregulation are tightly linked functions of responsive CD8^+^ T cells [r = 0.9135, 95% C.I. = 0.5861 to 0.9845, two tailed p value = 0.0015; Pearson correlation]. Thus, it is reasonable to infer CD8^+^ T cells that upregulate perforin are also producing new granzyme B.

**Figure 3:**
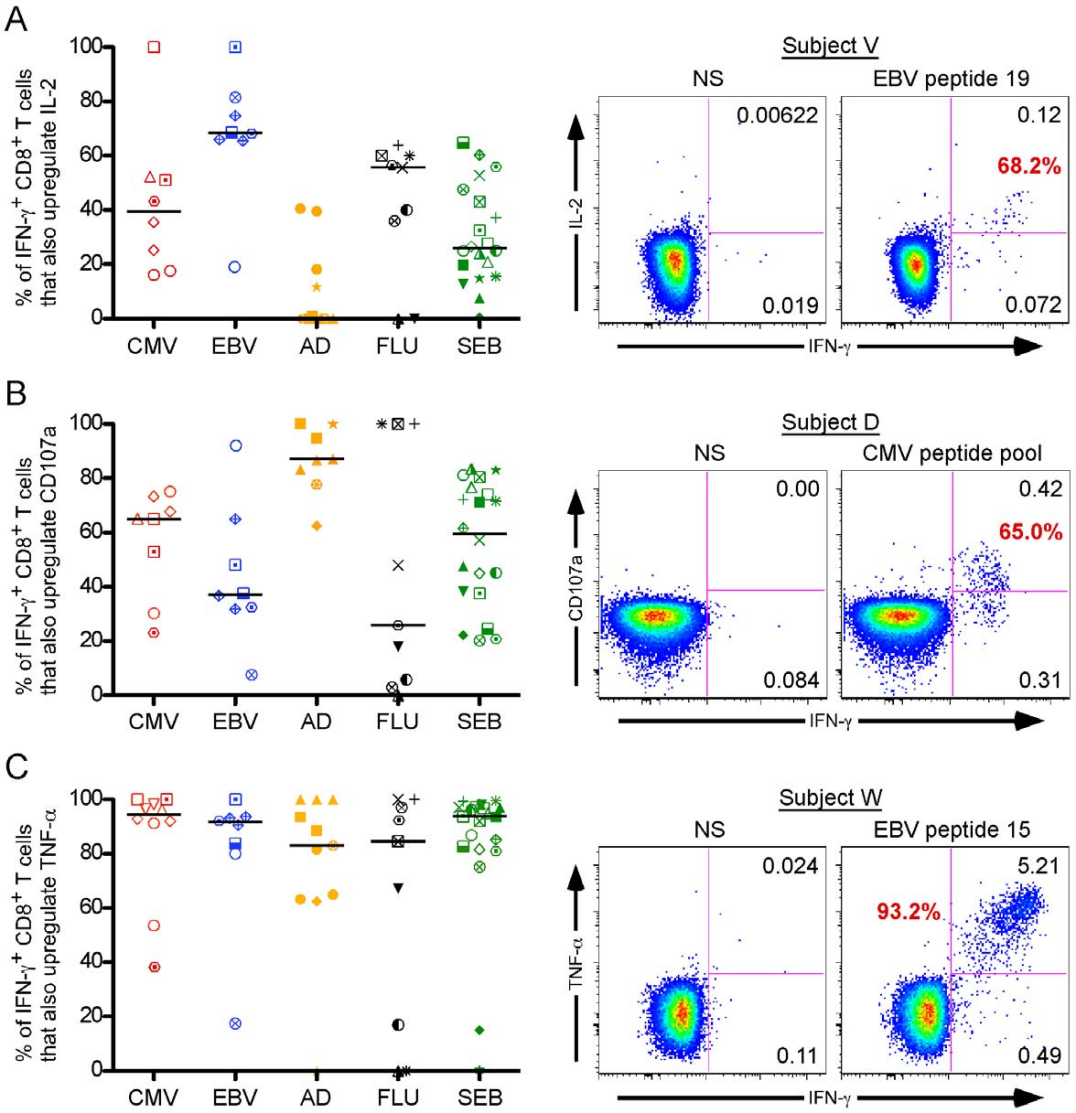
Individual CD8^+^ T cell functions do not predict universal viral control. IFN-γ producing virus-specific CD8^+^ T cells differentially upregulate IL-2 (A), degranulation (B), and TNF-α (C). The graphs (left panels) summarize the responses, stratified by virus: CMV responders represented in red, EBV in blue, Ad in orange, and flu in black. The black horizontal bars indicate the median response in each group. Representative staining results for each individual function are shown to the right of the graphs. Each function was quantified against IFN-γ to permit comparative analysis. The red number in each plot represents the proportion of IFN-γ producing CD8^+^ T cells that also produce that function, whereas the black numbers reflect the proportion of total CD8^+^ T cells that upregulate that function. NS =  no stimulation. Individual subject symbols are as described in Figure 1.

Next, we examined the capacity of virus-specific IFN-γ^+^ CD8^+^ T cells to also produce IL-2 (Figure 3A). In contrast to perforin, IL-2 production was elevated in EBV- and flu-specific CD8^+^ T cells [EBV: blue group, median = 71.7%, flu: black group, median = 55.7%, respectively], whereas in CMV and Ad it was much lower [CMV: red group, median (black bar) = 39.4%, Ad: orange group, median = 0.21%, respectively].

Finally, we analyzed degranulation capacity using the lysosomal and granule resident marker CD107a[29],[30], as well as TNF-α production [Figure 2B and 2C, respectively]. The CD107a response pattern across all the viral settings was similar to that of perforin: CMV and Ad stimuli induced strong CD8^+^ T cell degranulation [Figure 2B; CMV: red group, median (black bar) = 65.05%, Ad: orange group, median = 87.2%], whereas EBV and flu did to a lesser extent [EBV: blue group, median = 37.2%, flu: black group, median = 25.8%]. Unlike both CD107a and perforin, TNF-α production was ubiquitously expressed, as nearly every virus-specific IFN-γ^+^ CD8^+^ T cell also produced TNF-α [Figure 2C; CMV: red group, median (black bar) = 94.6%; EBV: blue group, median (black bar) = 91.35%; Ad: orange group, median (black bar) = 83.0%; flu: black group, median (black bar) = 84.6%].

Taken together, these results suggest that there are substantially different CD8^+^ T cell functional profiles against CMV, EBV, Ad, and flu, and that no single function (or pair of functions) likely defines a universal correlate of immune protection for all of these viruses.

### Polyfunctional profiles of virus-specific CD8^+^ T cell responses

**Figure 4:**
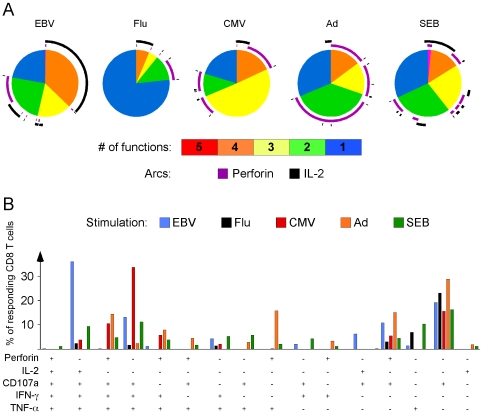
Rapid perforin upregulation and IL-2 production define distinct functional subsets for each model virus. (A) Average functional response profile of the 23 donors, symbolized as a pie chart, and stratified by virus. PBMC from each subject were simultaneously assayed for perforin, IFN-γ, IL-2, TNF-α, and CD107a upregulation. Pie slices represent the proportion of responding CD8^+^ T cells that upregulated all 5 (red), 4 (orange), 3 (yellow), 2 (green) and 1 (blue) function(s). Purple arcs denote the proportion of the responses that include perforin upregulation; black arcs denote responses that upregulate IL-2. (B) Distribution of responding CD8^+^ T cells across 16 different functional subsets. Possible functional combinations that were not observed are omitted for clarity. EBV responses are illustrated with blue bars, flu responses with black bars, CMV responses with red bars, Ad responses with orange bars, and SEB responses with green bars. P  =  Perforin, 2 = IL-2, 7 = CD107a, G  =  IFN-γ, T  =  TNF-α. The y-axis denotes the proportion of the total CD8^+^ T cell response to the given virus that produces a specific profile of functions.

We next characterized the polyfunctionality of the virus-specific CD8^+^ T cells from each donor to see if a particular response profile(s) was consistently detected in all viral contexts. We grouped donor responses according to viral specificity, and then assessed the average CD8^+^ T cell polyfunctional profile specific for that viral infection. As shown in Figure 4A, each viral antigen stimulated a unique functional profile consisting of varying degrees of polyfunctionality. Perforin production (designated by purple arcs around the pies) dominated the Ad-specific response profile compared to the other viral stimulations, and was highly expressed in a 4+ population (orange pie slice) together with CD107a, IFN-γ, and TNF-α. In the case of CMV, perforin upregulation was somewhat less dominant, but was similarly co-expressed with CD107a, IFN-γ, and TNF-α (Figure 4B) to form a substantial 4+ population (Figure 4A, orange pie slice). EBV also generated a highly multi-functional response, however the 4+ population (orange pie slice) was composed entirely of an IL-2^+^CD107a^+^IFN-γ^+^TNF-α^+^ CD8^+^ T cell subset (Figure 4B). IL-2 production also dominated the 4+ polyfunctional profile of flu, as the IL-2^+^CD107a^+^IFN-γ^+^TNF-α^+^ subset was again the principal multi-functional population (Figure 4B). In fact, as depicted by the arcs around the pies in Figure 4A, it appears that IL-2 production (black arcs) and perforin upregulation (purple arcs) generally are not co-expressed within any polyfunctional population. Thus, while every virus stimulated a high frequency of CD8^+^ T cells capable of four effector functions simultaneously, CMV and Ad induced a perforin driven 4+ responder population, whereas EBV and flu preferentially stimulated an IL-2 dominated 4+ subset.

### An inverse relationship exists between perforin and IL-2 upregulation

**Figure 5:**
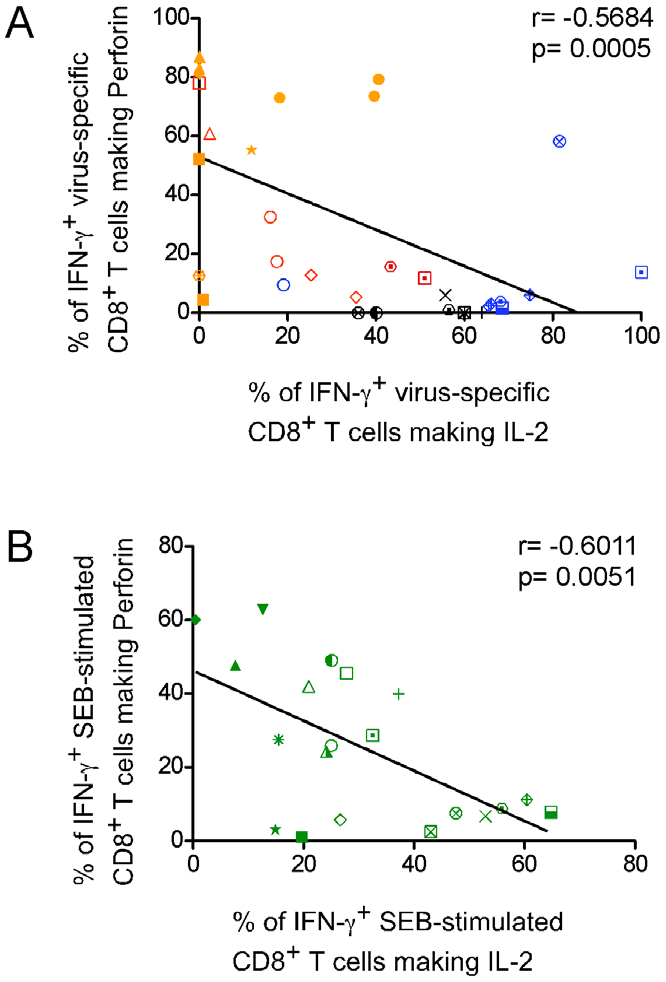
Rapid perforin upregulation and IL-2 production are inversely correlated functions of virus-specific CD8^+^ T cells. (A) The proportion of antigen-specific IFN-γ^+^ CD8^+^ T cells making new perforin was plotted against that of IFN-γ^+^ CD8^+^ T cells making IL-2. Responses from all donors for every virus were included. Individual symbols represent individual donors. CMV responses are shown in red, EBV in blue, Ad in orange, and flu in black. (B) Perforin^+^ IFN-γ^+^ or IL-2^+^ IFN-γ^+^ SEB-induced responses from each donor were plotted. The significance of the relationship was determined by calculating the Pearson product-moment correlation coefficient, r.

Strikingly, none of the virus-specific CD8^+^ T cell response profiles included a 5+ subset, suggesting that responding CD8^+^ T cells rarely upregulate perforin and IL-2 simultaneously. To further explore this possibility, we plotted the proportion of antigen-specific IFN-γ^+^ cells producing either new perforin or IL-2. As depicted in Figure 5A, a statistically significant inverse correlation exists between IL-2 and perforin positivity in virus-specific IFN-γ^+^ CD8^+^ T cells (r = −0.5684, 95% C.I. = −0.7604 to −0.2849, p<0.0005; Pearson correlation). A strong correlation also results if only SEB-induced responses are considered (Figure 5B; r = −0.6011, 95% C.I. = −0.8244 to −0.2159, p<0.0051; Pearson correlation). We performed our analysis on total CD8^+^ T cells, even though naïve cells preferentially produce IL-2 over IFN-γ and perforin. In our data set, however, the contribution of IL-2 from naïve CD8^+^ T cells in response to SEB stimulation is minimal compared to the antigen-experienced cells (not shown), and does not change the relationship we observe between IL-2 and perforin.

Thus, although not absolute, simultaneous production of IL-2 and perforin within the same CD8^+^ T cell, or within a virus-specific CD8^+^ T cell population, is exceptionally rare, suggesting a mutually exclusive relationship between these functions.

### The transcription factor T-bet preferentially accumulates in CD8^+^ T cells that rapidly upregulate perforin while those producing IL-2 express CD28

**Figure 6:**
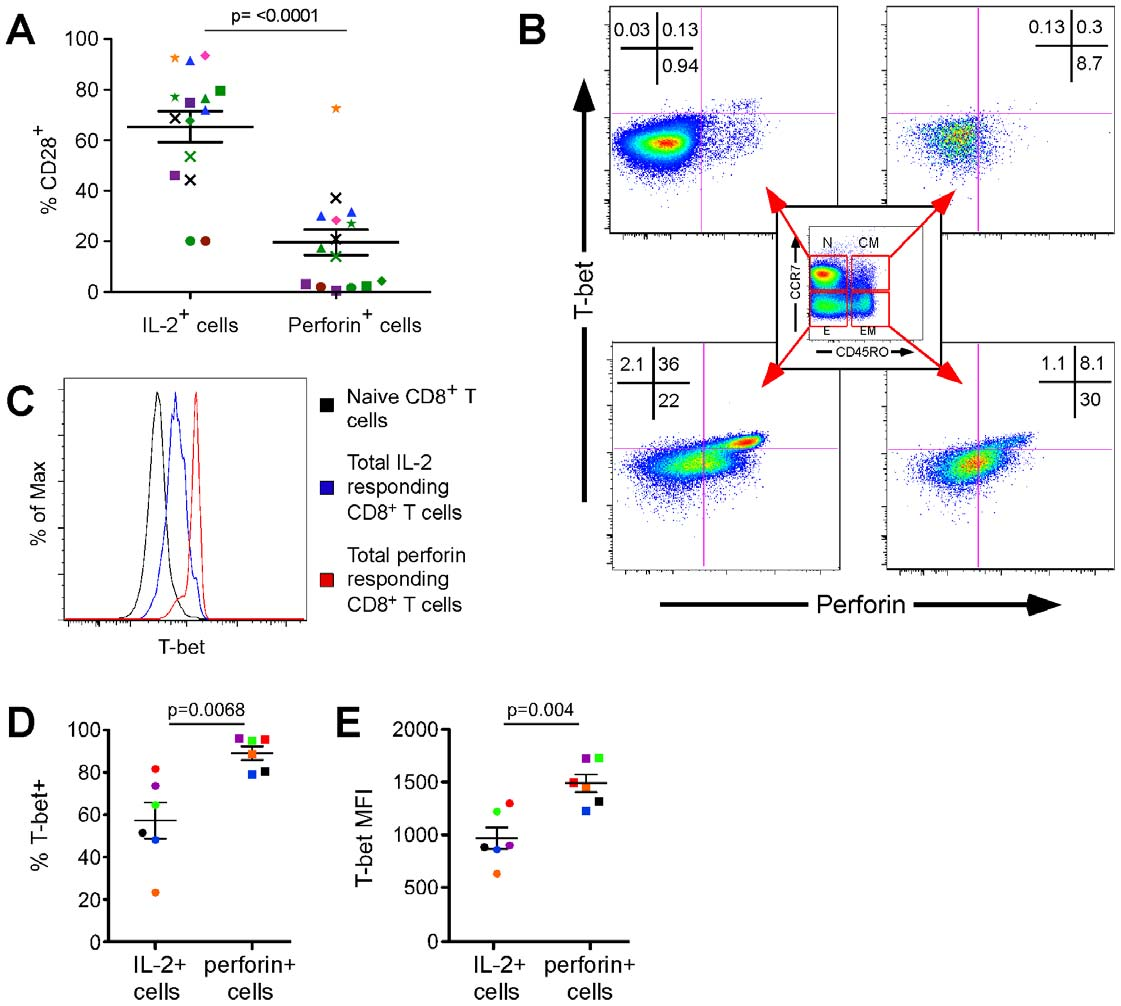
T-bet is preferentially expressed in CD8^+^ T cells that rapidly upregulate perforin and promotes an effector phenotype. (A) Proportion of perforin^+^ (right) and IL2^+^ (left) CD8^+^ T cells that express CD28. Observations are paired; PBMC from every subject (designated by a unique symbol) were activated using peptide stimuli (coloured) and SEB (green symbols). Statistical differences were determined by a Paired t test, two-tailed, t = 8.631, df = 14. (B) Levels of T-bet and perforin expression in resting naïve cells (N, CCR7^+^CD45RO^−^; upper left plot), central memory cells (CM, CCR7^+^CD45RO^+^; upper right plot), effector memory cells (EM, CCR7^−^CD45RO^+^; lower right plot), and effector cells (E, CCR7^−^CD45RO^−^; lower left plot). (C) Relative expression of T-bet in naïve (black line), IL-2 producing (blue line), and perforin upregulating (red line) CD8^+^ T cells stimulated with SEB. (D) Proportion of CD8^+^perforin^+^ (right, square symbols) and CD8^+^IL2^+^ (left, circle symbols) T cells that also express T-bet upon stimulation with SEB. Observations are paired and colour-coded by subject. Statistical difference determined by a Paired t test, two-tailed, t = 4.427, df = 5. (E) Abundance of T-bet on a per cell basis in CD8^+^perforin^+^ (right, square symbols) and CD8^+^IL2^+^ (left, circle symbols) T cells after SEB stimulation. Observations are paired and colour-coded by subject. Statistical difference determined by a Paired t test, two-tailed, t = 5.044, df = 5.

There are several precedents characterizing the functional attributes of particular CD8^+^ T cell memory phenotypes in the context of specific viral infections[31],[32],[33],[34], however the basis for these differences remains unknown. We investigated the potential role of two cellular factors in determining the preferential expression of either perforin or IL-2 by virus-specific human effector CD8^+^ T cells: CD28, a co-receptor whose signaling is critical for the induction of IL-2 production[35],[36], and T-bet, the T-box transcription factor associated with effector function[37],[38],[39]. As illustrated in Figure 6A, CD28 is commonly detected on IL-2 producing CD8^+^ T cells (mean = 65.4%, SEM = 6.125, 95% C.I. 52.2–78.5%), whereas its expression is significantly lower on those upregulating perforin (mean = 19.7%, SEM = 5.047, 95% C.I 8.85–30.5%; p = <0.0001, Paired t-test). Within all subjects tested (each represented by a unique symbol), CD28 expression was always higher on the antigen-specific CD8^+^ T cells (each stimuli represented by a unique colour) producing IL-2 than their counterparts upregulating perforin (Figure 6A). A phenotypic evaluation of IL-2 producing and perforin upregulating cells reveals that the former cells bear relatively high levels of CD27 and CD28 but low levels of CD57, whereas the latter cells are mostly CD27^+/−^CD28^lo^CD57^hi^ (Supplementary Figure S1). Thus, CD28, which is important mechanistically for IL-2 production, is not commonly detected on CD8^+^ T cells that are rapidly upregulating perforin.

Although T-bet has been linked to the development of T_H_1 responses and effector function in murine CD4^+^ and CD8^+^ T cells, respectively[38],[39], a similar relationship has yet to be formally demonstrated in humans. The only possible exception is a clinical study of ICOS-deficient sibling patients in whom impaired CD8^+^ T cell effector function and decreased development of memory T cell populations was indirectly linked to T-bet [40]. As illustrated in Figure 6B, we first examined the levels of T-bet in resting human CD8^+^ T cell memory subsets directly *ex vivo*. Effector cells (CCR7^−^CD45RO^−^), and to a lesser degree effector memory cells (CCR7^−^CD45RO^+^), exhibited concordant levels of perforin and T-bet, whereas both factors were absent in naïve (CCR7^+^CD45RO^−^) and central memory cells (CCR7^+^CD45RO^+^). We then stimulated the PBMC from the same donor with SEB to activate all the functional subsets and assessed T-bet expression in the fraction of CD8^+^ T cells that rapidly upregulates perforin, compared to that producing IL-2 (Figure 6C). T-bet expression was most pronounced in perforin-producing cells compared to both IL-2 producing cells and naïve CD8^+^ T cells, although IL-2 producing cells did harbor more T-bet than naïve CD8^+^ T cells. Overall, in 6 separate individuals we observed T-bet in a higher proportion of CD8^+^ T cells upregulating perforin than in those producing IL-2 (Figure 6D: median = 91.75% vs. 58.1%, mean = 89.05±7.77% vs. 57.25±20.93%, p = 0.0068, Paired t-test). Furthermore, CD8^+^ T cells that rapidly upregulate perforin express more T-bet on a per cell basis than those that produce IL-2 (Figure 6E: median = 1476 vs. 893.5, mean = 1491±205.9 vs. 967.2±248.7 median fluorescence intensity, p = 0.004, Paired t-test).

In conclusion, the preferential expression of the transcription factor T-bet in CD8^+^ T cells that rapidly upregulate perforin over those that produce IL-2 supports a significant role for T-bet in the differentiation of antigen-specific human CD8^+^ T cells into cytotoxic effector cells. Furthermore, the expression of CD28 co-receptor is correlated to IL-2 production.

## Discussion

What defines the “optimal” CD8^+^ T cell polyfunctional profile for viral infections in humans? The data we have presented here suggest that based upon the characteristics of replication, latency, persistence, and antigen load, every virus will potentially stimulate multiple polyfunctional profiles distinct from those of other viral infections. Here we examined four different viral infections, each of which is controlled or eliminated at least in part by viral-specific CD8^+^ T cells, and for each of these viral specificities we have found unique polyfunctional profiles. At the simplest level, it appears that rapid perforin upregulation and IL-2 production define complementary functional CD8^+^ T cell subsets that bear unique phenotypic profiles and predominate according to antigenic burden.

It has long been appreciated that CD8^+^ T cells play a pivotal role in the direct elimination of virally infected cells, and that perforin is a key mediator of this process through its distinct ability to enable the entry of apoptosis-inducing granzymes[17],[20]. We previously demonstrated that virus-specific CD8^+^ T cells rapidly upregulate perforin after activation and then target the protein directly to the interface between the CTL and its target[24]. This sustained production and targeted release of new perforin after stimulation may allow the CD8^+^ T cell to recognize and kill additional targets after the initial release and depletion of pre-formed perforin stored in cytotoxic granules. The measurement of new perforin production is significant because it serves as a gauge of a CD8^+^ T cells' potential to repeatedly eliminate infected host cells and, hence, control viral pathogenesis. Here we show that rapid perforin upregulation is a highly specialized ability not common to all CD8^+^ T cells. Rather, it appears to be tied to the antigenic history of the cell.

Whereas perforin upregulation and degranulation are commonly associated functions of CTL, there appears to be a mutually exclusive relationship between new perforin and IL-2 upregulation. Rapid perforin upregulation ability is not commonly observed against influenza and EBV. The fact that these pathogens establish latency (EBV) or are rapidly cleared (flu) suggests that antigen load or continual antigen exposure may in part maintain perforin upregulation ability and drive effector phenotype differentiation. For these viruses, proliferation and/or consequent differentiation of the EBV and flu-specific memory CD8^+^ T cells may be necessary to induce perforin upregulation. In contrast, perforin upregulation is more prominent in response to CMV and Adenovirus. CMV infection is characterized by continual low-level viral replication, and can induce massive expansions of CMV-specific effector CD8^+^ T cells[41]. Hence, it is not entirely surprising that CMV-specific CD8^+^ T cells should be capable of rapid perforin upregulation. The potent ability of Ad-specific CD8^+^ T cells to upregulate perforin, on the other hand, was unexpected since Ad, much like flu, should be rapidly cleared. However, there is evidence that Ad can become persistent[42]. Furthermore, there are at least 51 different Ad serotypes in circulation around the world, with differential levels of neutralizing antibodies against each serotype being present within a given individual[43]. As there is a high degree of sequence conservation between various Ad serotypes[26], cross-reactive CD8^+^ T cells are quite common, even between distantly related Ad serotypes[25]. Therefore, it is likely that exposure to Ad antigen from persisting virus or exposure to different Ad serotypes repeatedly activates Ad-specific CD8^+^ T cells, thereby driving the maintenance of a stable Ad-specific effector population. In contrast, EBV- and flu-specific CD8^+^ T cells typically produce IL-2 and bear a central memory phenotype. Since antigen load in the chronic phase of these infections is low or absent, the responding CD8^+^ T cell populations have likely differentiated to a resting memory state, where immediate cytotoxic potential is not critical.

An alternative interpretation of our data is that control of some viruses requires differential functional profiles: a polyfunctional response led by IL-2 is necessary for EBV and influenza, while CMV and Adenovirus may need to be controlled or cleared by a perforin-dominated response. Our phenotypic profiling of the perforin and IL-2 functional subsets as effector and central memory-like T cells, respectively, (Supplementary Figure S2) is in agreement with previous work on CD8^+^ T cell maturation, which included the measurement of pre-formed perforin, to ascribe discrete functional attributes to specific stages of differentiation[31],[33],[44],[45]. On this basis, several studies have related particular memory phenotypes to control of certain viral infections[32],[34],[46],[47],[48]. Our work elaborates on these earlier studies by correlating specific, complex functional profiles to immunity against different viral pathogens, irrespective of stage of differentiation. The D48 perforin antibody used here enabled the measurement of *both* pre-formed and new perforin, permitting a detailed characterization of the complete perforin compartment and a sharper definition of the mutually exclusive relationship between the perforin and IL-2 CD8^+^ T cell functional subsets. Given the cross-sectional nature of our study, it is not possible to ascertain whether new perforin and IL-2 dominated functional subsets represent stable CD8^+^ T cell populations that actually abrogate their respective viral burdens, or if they are subsets that result as a consequence to a waning antigenic presence. A longitudinal analysis of CD8^+^ T cells responding to the live yellow fever virus and smallpox vaccines recently showed that both vaccines generated a primary virus-specific CD8^+^ T cell response that passed through an obligate effector phase in which the cells abundantly expressed perforin and granzyme B[49]. The cells then differentiated into long-lived memory cells that maintained the ability to proliferate and secrete effector cytokines in response to antigen[49]. Thus, the perforin and IL-2 functional subsets we describe herein likely serve to mediate protective immunity at different stages of infection.

What is responsible for the transition from a polyfunctional response highlighted by rapid perforin upregulation to an IL-2-dominated response? What determines the array of functions a CD8^+^ T cell can perform? Antigen sensitivity has recently been reported to be required for the development of a polyfunctional CD8^+^ T cell response[50], but the mechanism behind this phenomenon remains to be elucidated. Our association between elevated CD28 levels and IL-2 production by antigen-specific CD8^+^ T cells confirms published findings describing a direct role for CD28 signaling in IL-2 induction[35],[36]. Our observation that new perforin preferentially accumulates in human CD8^+^ T cells that express the transcription factor T-bet supports the role of T-bet as a ‘master regulator’ of effector CD8^+^ T cell responses[37],[38],[51],[52]. Corollary, the relatively reduced levels of T-bet in the IL-2 producing CD8^+^ T cells supports data from mouse models of T cell differentiation which demonstrate that T-bet is also a transcriptional repressor of IL-2[53],[54]. Furthermore, T-bet expression correlates with the development of short-lived effector cells in mice, whereas a moderate decrease in T-bet expression promotes long-lived memory [51],[52],[55]. Thus, our data suggest that T-bet is intimately involved in determining the functional capabilities of virus-specific CD8^+^ T cells, and provide an important premise in humans on which to explore the relationship between T-bet and the perforin gene.

The interplay between IL-2 and perforin thus necessitates a re-evaluation of our current interpretation of CD8^+^ T cell polyfunctionality. The prevailing rationale is that antigen-specific polyfunctional CD8^+^ T cell responses containing IL-2 are most effective at controlling viral replication[13]; a premise that is driving current T cell based vaccine strategies. Our data suggest that we need to reclassify CD8^+^ T cell polyfunctionality into at least two distinct types: polyfunctional memory (IL-2 + IFN-γ + other functions without perforin) or polyfunctional effector (perforin + IFN-γ + other functions without IL-2), each profile being distinct and worthy of independent consideration. In reality, *both* functional subsets will likely be required for a protective immune response, each being instrumental at different stages of infection.

## Materials and Methods

### Ethics statement

The University of Pennsylvania's Center for AIDS Research Human Immunology Core (IRB# 705906), The Wistar Institute (IRB#2506215), and Duke University (IRB exempt) obtained written, informed consent from every donor subject in order to collect PBMC samples and approved the methods employed in this study.

### Cells and peptides

PBMC were cryopreserved in fetal bovine serum (FBS; ICS Hyclone, Logan, Utah) containing 10% dimethyl sulfoxide (DMSO; Fisher Scientific, Pittsburgh, Pennsylvania). Individual peptide stimuli were determined by prior epitope mapping by IFN-γ Elispot experiments. In subjects for whom epitopes were not identified, pools of peptides (15mers overlapping by 11 amino acids) were used. Regarding the use of 15 versus 9 amino acid individual peptides, several studies have shown that although some variation in function and magnitude can be present between some epitopes, on average the magnitude and functionality of responses to CTL epitopes represented as a 9 mer or within a 15 mer peptide are generally equivalent. As a proof of concept, we stimulated Subject E with both an optimal and a 15 amino acid peptide containing the epitope TPRVTGGGA and quantified very similar responses (Supplemental Figure S3).

### Antibodies

Antibodies for surface staining included anti-CD4 PE Cy5-5 (Invitrogen; Carlsbad, California), anti-CD107a FITC (BD Biosciences; San Jose, California), anti-CD8 Qdot 655 (custom) or TRPE (Invitrogen; Carlsbad, California), anti-CD14 Pac Blue (BD Biosciences; San Jose, California), anti-CD16 Pac Blue (BD Biosciences; San Jose, California), and anti-CD19 Pac Blue (Invitrogen; Carlsbad, California), anti-CD57 Qdot 565 (custom), anti-CD27 PE Cy5 (Beckman Coulter, Inc; Fullerton, California) or PerCP Cy5-5 (Biolegend; San Diego, California), anti-CD28 ECD (Beckman Coulter, Inc; Fullerton, California) and anti-CD45RO Qdot 605/705 (custom) or ECD (Beckman Coulter, Inc; Fullerton, California). Antibodies for intracellular staining included anti-CD3 Qdot 585 (custom), anti-Granzyme B Texas Red PE (BD Pharmingen; San Diego, California), anti-IFN-γ Alexa 700 (BD Pharmingen; San Diego, California), anti-IL-2 APC (BD Pharmingen; San Diego, California), anti-TNF-α PE Cy7 (BD Biosciences; San Jose, California), and anti-T-bet (Santa Cruz Biotechnology; Santa Cruz, California). Custom conjugations to Quantum (Q) dot nanocrystals were performed in our laboratory as previously described[56], with reagents purchased from Invitrogen (Carlsbad, California). Anti-human perforin antibodies were purchased from Tepnel (clone D48, Besancon, France) and BD Biosciences (clone δG9, San Jose, California).

### FACS staining assay

Cryopreserved PBMC were thawed, and then rested overnight at 37°C, 5% CO_2_ in complete medium [RPMI (Mediatech Inc; Manassas, Virginia) supplemented with 10% FBS, 1% L-glutamine (Mediatech Inc; Manassas, Virginia), and 1% penicillin-streptomycin (Lonza; Walkersville, Maryland), sterile filtered] at a concentration of 2×10^6^ cells per ml medium in 12-well plates. The next day, the cells were washed with complete medium and resuspended at a concentration of 1×10^6^ cells/ml with costimulatory antibodies (anti-CD28 and anti-CD49d; 1 µg/ml final concentration; BD Biosciences; San Jose, California), in the presence of monensin (0.7 µg/ml final concentration; BD Biosciences; San Jose, California) and brefeldin A (1 µg/ml final concentration; Sigma-Aldrich; St. Louis, Missouri). Anti-CD107a was always added at the start of all stimulation periods, as described previously[29]. As a negative control, 5 µl of DMSO was added to the cells, an equivalent concentration compared to the peptide stimulus. SEB served as the positive control (1 µg/ml final concentration; Sigma-Aldrich; St. Louis, Missouri). Peptide stimulations were performed at a final concentration of 2 µM. Stimulation tubes were incubated at 37°C, 5% CO_2_ for six hours, after which cells were washed once with PBS and then stained for viability with Aqua amine-reactive viability dye (Invitrogen; Carlsbad, California) for ten minutes in the dark at room temperature. A cocktail of antibodies was then added to the cells to stain for surface markers for an additional twenty minutes. The cells were washed with PBS containing 1% bovine serum albumin (BSA, Fisher Scientific; Pittsburgh, Pennsylvania) and 0.1% sodium azide (Fisher Scientific; Pittsburgh, Pennsylvania), and permeabilized using the Cytofix/Cytoperm kit (BD Biosciences; San Jose, California) according to the manufacturer's instructions. A cocktail of antibodies against intracellular markers was then added to the cells and allowed to incubate for one hour in the dark at room temperature. The cells were then washed once with Perm Wash buffer (BD Biosciences; San Jose, California) and fixed in PBS containing 1% paraformaldehyde (Sigma-Aldrich; St. Louis, Missouri). Fixed cells were stored in the dark at 4°C until the time of collection.

### Flow cytometric analysis

For each specimen, between 500,000 and 1,000,000 total events were acquired on a modified flow cytometer (LSRII; BD Immunocytometry Systems; San Jose, California) equipped for the detection of 18 fluorescent parameters. Antibody capture beads (BD Biosciences; San Jose, California) were used to prepare individual compensation tubes for each antibody used in the experiment. Data analysis was performed using FlowJo version 8.7.3 (TreeStar, Ashland, Oregon). Reported data have been corrected for background.

### Figures

Canvas software, version 10.4.9 (ACD Systems; Miami, Florida), and Prism software, version 5.0 (Graphpad; La Jolla, California), were used to create the figures. Labels and boxes were added to raw data images in Canvas. The dots for Subject C in the bottom right panel of Figure 6 were enlarged in Canvas to facilitate visual identification and discrimination.

### Statistical analyses

Correlation between %IL-2 and %perforin of IFN-γ producing CD8^+^ T cells was determined by a two-tailed Pearson correlation test. A two-tailed Paired t-test was used to define statistically significant differences in CD28 and T-bet expression between IL-2 and perforin producing CD8^+^ T cells. Both analyses were performed using Prism software.

## Supporting Information

Figure S1 Differential Expression of CD28 on IL-2 and Perforin Upregulating CD8^+^ T cells. Donor PBMC were stimulated for 6 hours with peptide and/or SEB to induce perforin and IL-2 upregulation in order to assess the patterns of CD27, CD28, and CD57 expression on the activated cells. Shown above are 2 representative examples: Donor 317 developed a robust perforin response whereas Donor 232 mounted a strong IL-2 response as a result of SEB stimulation. For each subject, the dot plot on the left illustrates the total perforin or IL-2 response by the complete CD8^+^ T cell compartment, whereas the smaller dot plots on the right illustrate the expression of the cell surface markers on the responding (boxed) populations.
(0.16 MB PDF)

Figure S2 IL-2 and Perforin Upregulating CD8^+^ T cells Bear Different Memory Phenotypes. Top row: EBV peptide 19 specific response by Subject V. The dot plot on the left illustrates the distribution of all functional cells (blue dots), irrespective of function, among the entire CD8^+^ T cell population (black density plots), separated according to CD27 and CD45RO, whereas the blue dots in the right panel signify only IL-2 producing cells. Middle row: CMV peptide 21 specific response by Subject E. Left overlay dot plot shows all responding CD8^+^ T cells (red dots) whereas the right plot displays only perforin-upregulating cells (red dots). Bottom row: CMV peptide 21 specific response by Subject C. Left overlay dot plot illustrates the distribution of all responding CD8^+^ T cells (red dots) across the entire CD8^+^ T cell population (grey density plots), separated according to CD27 and CD45RO. The right plot displays both the IL-2 producing (blue dots) and perforin-upregulating (red dots) cells. The dots were enlarged to facilitate visual identification and discrimination.
(0.20 MB PDF)

Figure S3 The optimal peptide and the 15 amino acid peptide stimulate similar reponses. Donor E PBMC were stimulated for 6 hours with either the optimal length peptide representing the CMV pp65 epitope TPRVTGGGA or the longer 15 amino acid peptide that includes the CMV pp65 epitope: RKTPRVTGGGAMAGA. As illustrated above, both peptides induced similar IFN-γ, IL-2, and perforin repsonses from the CD8^+^ T cell compartment.
(0.19 MB PDF)
