# Supplementary material for: Perforin and IL-2 Upregulation Define Qualitative Differences among Highly Functional Virus-Specific Human CD8+ T Cells
Source: PLoS Pathog. 2010 Mar 5;6(3):e1000798. doi: 10.1371/journal.ppat.1000798 (PMC2832688; doi:10.1371/journal.ppat.1000798)
Supplement: Figure S1 — Differential Expression of CD28 on IL-2 and Perforin Upregulating CD8+ T cells. Donor PBMC were stimulated for 6 hours with peptide and/or SEB to induce perforin and IL-2 upregulation in order to assess the patterns of CD27, CD28, and CD57 expression on the activated cells. Shown above are 2 representative examples: Donor 317 developed a robust perforin response whereas Donor 232 mounted a strong IL-2 response as a result of SEB stimulation. For each subject, the dot plot on the left illustrates the total perforin or IL-2 response by the complete CD8+ T cell compartment, whereas the smaller dot plots on the right illustrate the expression of the cell surface markers on the responding (boxed) populations. (0.16 MB PDF) [file ppat.1000798.s001.pdf]

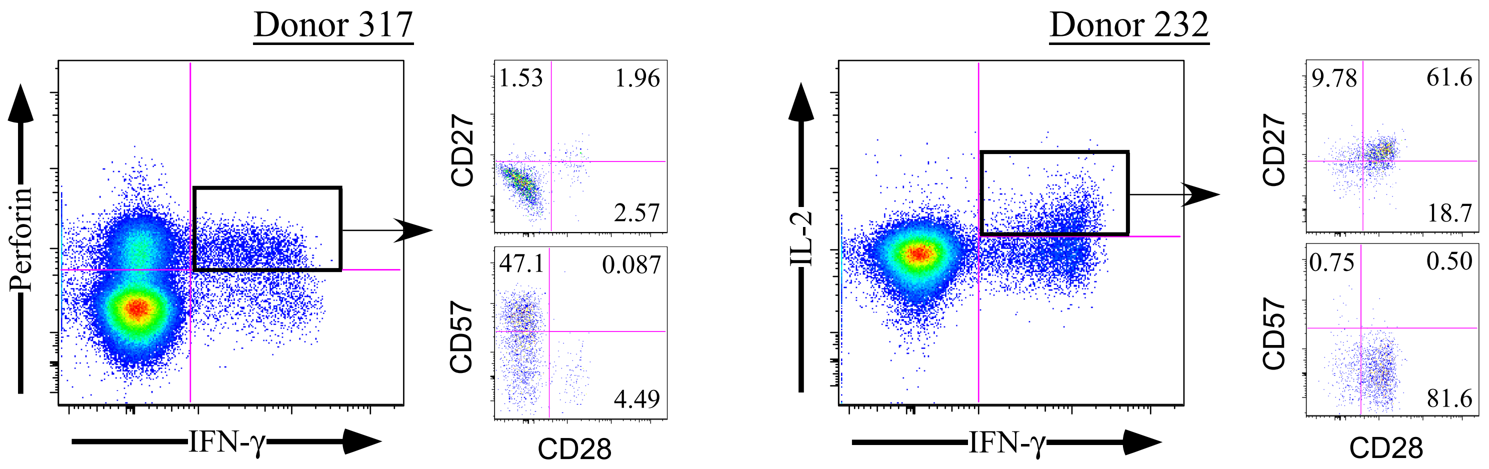


**Supplementary figure S1: Differential Expression of CD28 on IL-2 and Perforin Upregulating CD8+ T cells.** Donor PBMC were stimulated for 6 hours with peptide and/or SEB to induce perforin and IL-2 upregulation in order to assess the patterns of CD27, CD28, and CD57 expression on the activated cells. Shown above are 2 representative examples: Donor 317 developed a robust perforin response whereas Donor 232 mounted a strong IL-2 response as a result of SEB stimulation. For each subject, the dot plot on the left illustrates the total perforin or IL-2 response by the complete CD8+ T cell compartment, whereas the smaller dot plots on the right illustrate the expression of the cell surface markers on the responding (boxed) populations.
