# Supplementary material for: Perforin and IL-2 Upregulation Define Qualitative Differences among Highly Functional Virus-Specific Human CD8+ T Cells
Source: PLoS Pathog. 2010 Mar 5;6(3):e1000798. doi: 10.1371/journal.ppat.1000798 (PMC2832688; doi:10.1371/journal.ppat.1000798)
Supplement: Figure S2 — IL-2 and Perforin Upregulating CD8+ T cells Bear Different Memory Phenotypes. Top row: EBV peptide 19 specific response by Subject V. The dot plot on the left illustrates the distribution of all functional cells (blue dots), irrespective of function, among the entire CD8+ T cell population (black density plots), separated according to CD27 and CD45RO, whereas the blue dots in the right panel signify only IL-2 producing cells. Middle row: CMV peptide 21 specific response by Subject E. Left overlay dot plot shows all responding CD8+ T cells (red dots) whereas the right plot displays only perforin-upregulating cells (red dots). Bottom row: CMV peptide 21 specific response by Subject C. Left overlay dot plot illustrates the distribution of all responding CD8+ T cells (red dots) across the entire CD8+ T cell population (grey density plots), separated according to CD27 and CD45RO. The right plot displays both the IL-2 producing (blue dots) and perforin-upregulating (red dots) cells. The dots were enlarged to facilitate visual identification and discrimination. (0.20 MB PDF) [file ppat.1000798.s002.pdf]

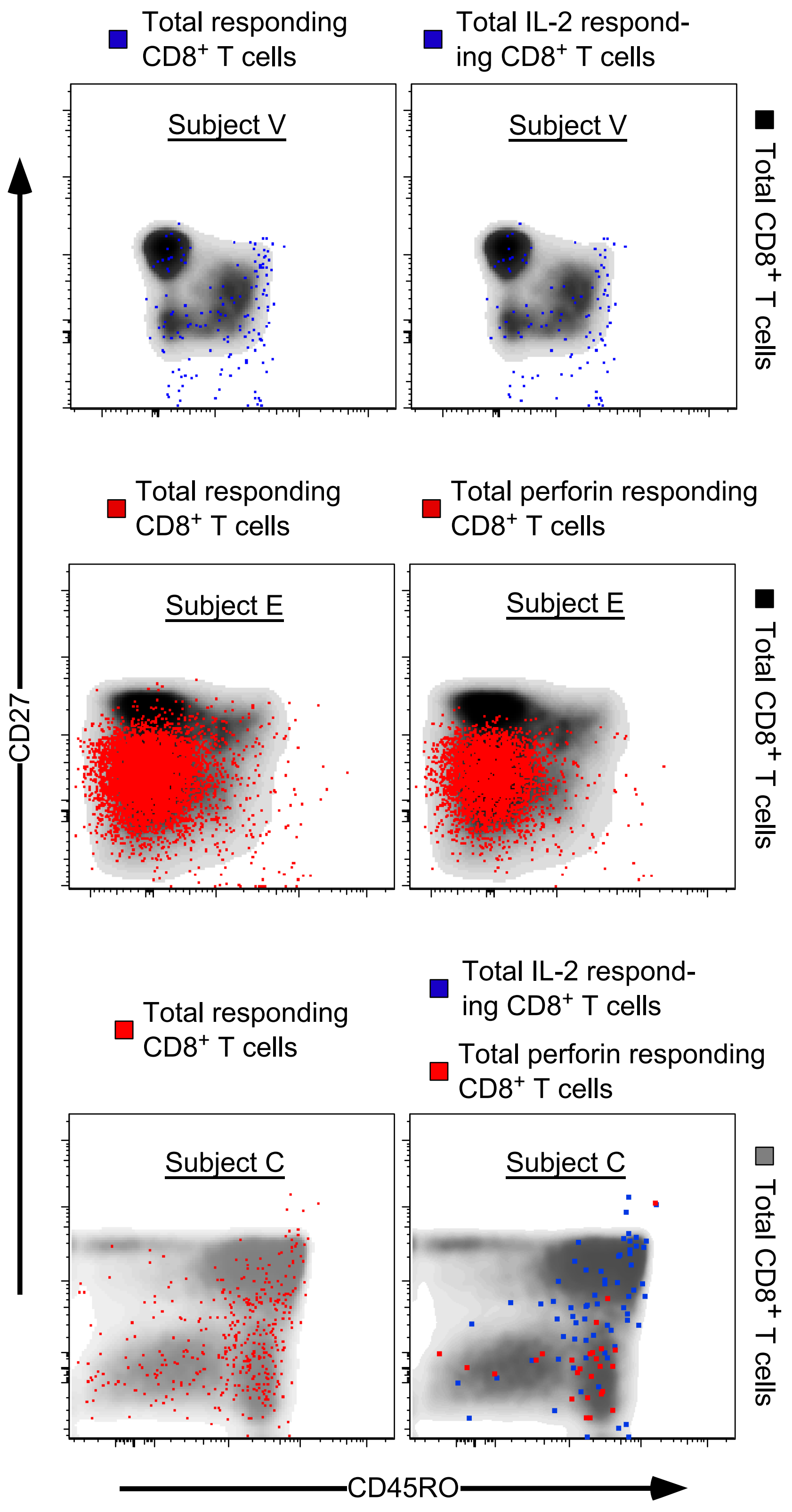

Supplementary figure S2: IL-2 and Perforin Upregulating CD8+ T cells Bear Different Memory Phenotypes. *Top row*: EBV peptide 19 specific response by Subject V. The dot plot on the left illustrates the distribution of all functional cells (blue dots), irrespective of function, among the entire CD8+ T cell population (black density plots), separated according to CD27 and CD45RO, whereas the blue dots in the right panel signify only IL-2 producing cells. *Middle row*: CMV peptide 21 specific response by Subject E. Left overlay dot plot shows all responding CD8+ T cells (red dots) whereas the right plot displays only perforin-upregulating cells (red dots). *Bottom row*: CMV peptide 21 specific response by Subject C. Left overlay dot plot illustrates the distribution of all responding CD8+ T cells (red dots) across the entire CD8+ T cell population (grey density plots), separated according to CD27 and CD45RO. The right plot displays both the IL-2 producing (blue dots) and perforin-upregulating (red dots) cells. The dots were enlarged to facilitate visual identification and discrimination.
