# Supplementary material for: Perforin and IL-2 Upregulation Define Qualitative Differences among Highly Functional Virus-Specific Human CD8+ T Cells
Source: PLoS Pathog. 2010 Mar 5;6(3):e1000798. doi: 10.1371/journal.ppat.1000798 (PMC2832688; doi:10.1371/journal.ppat.1000798)
Supplement: Figure S3 — The optimal peptide and the 15 amino acid peptide stimulate similar reponses. Donor E PBMC were stimulated for 6 hours with either the optimal length peptide representing the CMV pp65 epitope TPRVTGGGA or the longer 15 amino acid peptide that includes the CMV pp65 epitope: RKTPRVTGGGAMAGA. As illustrated above, both peptides induced similar IFN-γ, IL-2, and perforin repsonses from the CD8+ T cell compartment. (0.19 MB PDF) [file ppat.1000798.s003.pdf]

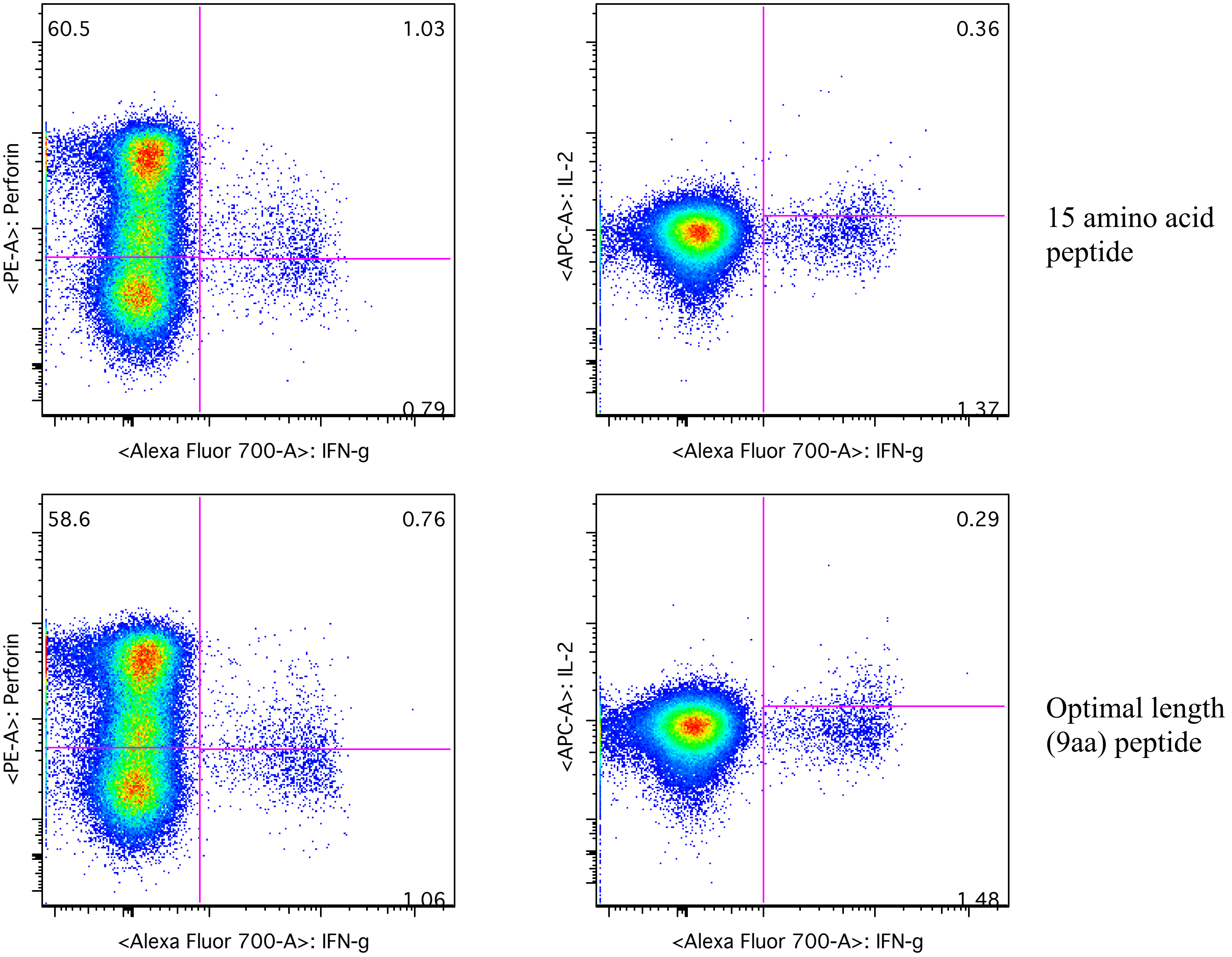

Supplementary figure S3: The optimal peptide and the 15 amino acid peptide stimulate similar reponses. Donor E PBMC were stimulated for 6 hours with either the optimal length peptide representing the CMV pp65 epitope TPRVTGGGA or the longer 15 amino acid peptide that includes the CMV pp65 epitope: RKTPRVTGGGAMAGA. As illustrated above, both peptides induced similar IFN-γ, IL-2, and perforin repsonses from the CD8+ T cell compartment.
